# Angiogenesis Inhibitors for the Treatment of Hepatocellular Carcinoma

**DOI:** 10.3389/fphar.2016.00428

**Published:** 2016-11-09

**Authors:** Massimiliano Berretta, Luca Rinaldi, Fabrizio Di Benedetto, Arben Lleshi, Vallì De Re, Gaetano Facchini, Paolo De Paoli, Raffaele Di Francia

**Affiliations:** ^1^Department of Medical Oncology, National Cancer InstituteAviano, Italy; ^2^Department of Medical, Surgical, Neurological, Metabolic and Geriatric Sciences, Second University of NaplesNaples, Italy; ^3^Liver and Multivisceral Transplant Center, University of Modena and Reggio EmiliaModena, Italy; ^4^Bioimmunotherapy of Human Cancers Unit, Centro di Riferimento Oncologico (CRO) National Cancer InstituteAviano, Italy; ^5^Division of Medical Oncology, Department of Uro-Gynaecological Oncology, Istituto Nazionale Tumori “Fondazione G. Pascale”–IRCCS NaplesNaples, Italy; ^6^Scientific Directorate, Centro di Riferimento Oncologico (CRO) National Cancer InstituteAviano, Italy; ^7^Department of Hematology, Istituto Nazionale Tumori “Fondazione G. Pascale”–IRCCS NaplesNaples, Italy

**Keywords:** neo-angiogenesis, hepatocellular carcinoma, treatment, target therapy, inhibitors and toxicity, pharmacogenomics

## Abstract

**Background**: Angiogenesis inhibitors have become an important therapeutic approach in the treatment of hepatocellular carcinoma (HCC) patients. The therapeutic inhibition of angiogenesis of Sorafenib in increasing overall survival of patients with HCC is a fundamental element of the treatment of this disease. Considering the heterogeneous aspects of HCC and to boost therapeutic efficacy, prevail over drug resistance and lessen toxicity, adding antiangiogenic drugs to antiblastic chemotherapy (AC), radiation therapy or other targeted drugs have been evaluated. The matter is additionally complicated by the combination of antiangiogenesis with further AC or biologic drugs. To date, no planned approach to understand which patients are more responsive to a given type of antiangiogenic treatment is available.

**Conclusion**: Large investments in the clinical research are essential to improve treatment response and minimize toxicities for patients with HCC. Future investigations will need to focus on utilizing patterns of genetic information to classify HCC into groups that display similar prognosis and treatment sensitivity, and combining targeted therapies with AC producing enhanced anti-tumor effect. In this review the current panel of available antiangiogenic therapies for the treatment of HCC have been analyzed. In addition current clinical trials are also reported herein.

## Introduction

The growth of cancers and the development of metastasis depend on angiogenesis, that is the adequate structure for blood supply, (Bruix et al., 2014a; Kubo et al., 2016). The process of angiogenesis consists of multiple, and mutually dependent steps. It begins with local degeneration of the basement membrane near capillaries, then by the invasion of the nearby stroma by the primary endothelial cells in the direction of the angiogenic stimuli. Endothelial cells migration is followed by the increase of endothelial cells, organized into 3D structures joining with new analogous structures forming a system of new blood vessels.

This is sometimes referred to as sprouting angiogenesis, which is a local process. In addition, endothelial cells can be generated not only by the division of pre-existing differentiated endothelial cells but also by the influx of circulating endothelial progenitor cells from bone marrow, a process sometimes referred to as systemic vasculogenesis (Kubo et al., 2016).

The beginning of angiogenesis affects the equlibrium between pro-angiogenic and antiangiogenic molecules in the local tissue microenvironment (Bruix et al., 2014a; Garbuzenko et al., 2016; Kubo et al., 2016). These molecules mediate multiple steps in the process of angiogenesis by selectively altering the characteristics of endothelial cells and associated perivascular structures (i.e., pericytes, vascular smooth muscle cells). Angiogenesis can occur by either emergent or non-emergent processes (Garbuzenko et al., 2016). Emergent angiogenesis implicates the division of new small vessels from pre-existing vessels. Non-emergent angiogenesis results from the enlargement, splitting, and fusion of pre-existing vessels produced by the proliferation of endothelial cells within the wall of a vessel. Trans-vascular bridges are occasionally observed in enlarged vessels produced by non-sprouting angiogenesis (Garbuzenko et al., 2016). This type of angiogenesis can occur concurrently with sprouting angiogenesis in the vascularization of organs or tissues. The mechanism of non-sprouting angiogenesis was found in brain metastases, and vascular endothelial growth factor (VEGF), also called vascular permeability factor (VPF) (Mazzaferro et al., 2014; Kim et al., 2015; Zampino et al., 2015), has a pivotal role in developmental, physiologic and pathologic neo-vascularization.

Vascular endothelial growth factor is a homodimeric heparin-binding glycoprotein that exists in at least four isoforms. The isoforms are subdivided in VEGF_121_, VEGF_165_, VEGF_189_, and VEGF_205_ according to the number of amino acids that each protein has.

There are at least four other members of the VEGF family. The aforementioned VEGF is referred to as VEGF-A. VEGF-B is believed to play a fundamental role in vasculogenesis; it may have also other roles like the activation of enzymes with invasive action on endothelial cells (Cervello et al., 2012; Mazzaferro et al., 2014). VEGF-C is frequently associated with lymph-angiogenesis, but more recently, its expression has been related with tumor angiogenesis in several systems. The role of VEGF-D is not so defined, but it may attach to VEGF receptor-2 (VEGFR-2) and VEGFR-3, and may induce *in vivo* angiogenesis. Little is known about VEGF-E, except that it binds to VEGFR-2 and can induce endothelial cell mitosis and angiogenesis (Cervello et al., 2012). VEGF receptors are expressed on endothelial cells and some tumor cells (Mazzaferro et al., 2014).

Among these molecules, VEGF is the most relevant factor for maintaining tumor cells growth and mediates its activity by specific receptors, called VEGF receptors (VEGFRs). VEGF is the target of numerous anti-cancer medications helpful in the management of many cancers including colon cancer, ovarian cancer, and glioblastoma multiforme (Korpanty and Smyth, 2012; Falchook et al., 2013; Ellingson et al., 2014). The primary angiogenic receptors for VEGF is VEGFR-1 and VEGFR-2. VEGFR-1 and VEGFR-2 are expressed in some types of vascular endothelial and cancer cells. VEGFRs are part of the tyrosine kinase receptors (TKr) family. VEGFR consists in seven extracellular immunoglobulin-like domains containing the tyrosine kinase domain (TKD). The binding between VEGF and its receptor origins the VEGFRs' dimerization, leading to the activation of intrinsic TK (Koch et al., 2011). Different VEGFRs create different signals, regardless of the elevated homology within the TKD. VEGFR-1 as a regulatory role in angiogenesis is scarcely autophosphorylated by VEGF in endothelial cells.

**Figure 1:**
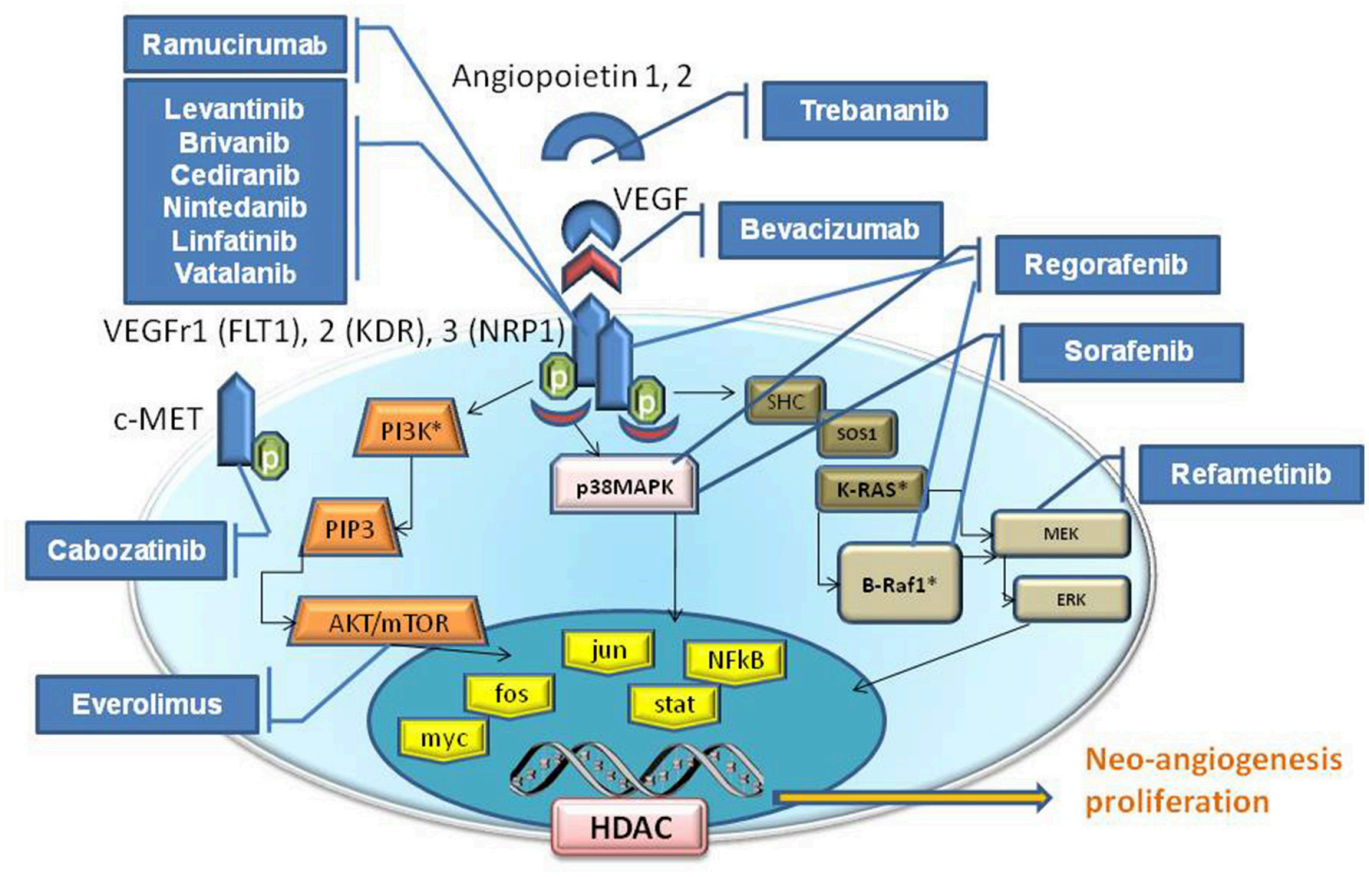
**Schematic signaling pathways elicited by VEGF**. The Tyrosine kinase proteins, Serine/Threonine Kinase (AKT etc), and GTPase (K-Ras) pathways are illustrated inside the cytoplasm compartment. The drugs (blu boxes) are indicative for their target that blocks/inhibit their effect on neo-angiogenesis, cell proliferation, apoptosis and etc. VEGFR family is composed by VEGFR1 (Alias FLT1), VEGFR2 (Alias KDR), VEGFR3 (Alias NRP1). Receptors for growth factors (VEGFR, FGFR, PDGFR) activate intracellular receptor tyrosine kinases (RTKs) and the downstream RAS/RAF/mitogen-activated protein extracellular kinase (MEK)/extracellular signal-regulated kinase (ERK) signaling pathway, and promote the growth, migration and morphogenesis of vascular endothelial cells, thus increasing vascular permeability by activating nuclear proteins (yellow boxes). Angiopoietin 1, 2 (Tie1,2) and VEGF are the principal angiogenic growth factors.

In addition to TKr pathways, when considering growth factors such as VEGF, discussing signaling events that are activated by cell-cell or cell-matrix interactions are highly important. Noteworthy is the crucial role of the downstream RAS/RAF/mitogen-activated protein extracellular kinase (MEK)/extracellular signal-regulated kinase (ERK) signaling pathway, and activated Rac1 (Wang and Hartnett, 2016). The new angiogenesis inhibitor agents target these alternative signaling pathway (Figure 1).

Nevertheless, current clinical trials have demonstrated that VEGFR-1, as a fundamental mediator of both physiologic and developmental angiogenesis may direct to the aggressive actions of HCC cells (Yi et al., 2011). VEGFR-2, mediating the development of endothelial cells and their permeability to cells and molecules upon binding of VEGF is directly implicated in angiogenesis process (Shibuya and Claesson-Welsh, 2006). A preclinical study in murine has exposed that the combination of anti-VEGFR-1 and anti-VEGFR-2 molecules might efficiently inhibit the expansion of HCC (Yoshiji et al., 2004). Current clinical trials are reported in this review.

Thus far, agents that target VEGF ligand-receptor system have been reported to have mild toxicity in clinical trials compared to that of standard chemotherapy; on the other hand, adverse effects have been reported (i.e., hypertension, nausea, headaches, thrombotic events, and proteinuria). The long-term effects of antiangiogenic therapy are yet unidentified.

### Background of hepatocellular carcinoma

HCC is the most frequent primary cancer of the liver and is the 5th and the 3rd leading cause of death from cancer worldwide in women and men, respectively (Ursino et al., 2012). Historically, the best known risk factors are represented by HBV, HCV virus infection (Berretta et al., 2015) and alcohol abuse with the resulting complications. Despite the potential decline of HCV cirrhosis in the coming years to the advent of new antiviral therapies, the increase of metabolic and NASH, constitute additional elements of risk (Thomas, 1996; Risau, 1997). A range of therapies are used in the management of HCC; the best outcomes are seen in patients who fulfill the criteria to undergo liver transplantation, surgical resection or loco regional therapies including transarterial chemoembolization (TACE), radiofrequency ablation and percutaneous ethanol injection (Senger et al., 1983; Ferrara et al., 2003; D'Amico et al., 2015a). More recently, the identification of cellular pathways playing a key role in the pathogenesis of HCC, primarily neoangiogenesis, has led to development of targeted drugs (Veikkola et al., 2000; Canzonieri et al., 2015) (Figure 1). Recent studies have described several mutational profile in HCC (Cervello et al., 2012; Falchook et al., 2013; Mazzaferro et al., 2014). Deep-sequencing studies have confirmed that promoter region of TP53 and β-Catenin 1 (CTNNB1) are frequently mutated in about 33 and 22% of HCC patients, respectively (Hirotsu et al., 2016). Mutations in these genes are mutually exclusive, an indication that they could act as drivers of tumor progression (Zhu et al., 2006; Sun et al., 2011). Overall, signaling pathways and apoptosys are altered in the multistep process of liver carcinogenesis. Sorafenib is a multi-targeted TK inhibitor, which is the only systemic agent found to increase survival time in patients with locally advanced and/or metastatic HCC (Berretta et al., 2013). In particular, it has been shown to improve survival by 2.3–2.8 month and currently represents the standard of care for these patients (Santoro et al., 2013). Novel targeted drugs are in the development stage but clinical trials have not provided satisfactory results to date.

VEGF is over expressed in HCC and is correlated with poorer clinical outcomes. This suggests that VEGF-mediated signaling is fundamental in HCC pathogenesis and it is a therapeutic target (Zhu et al., 2011).

### Can we improve angiogenesis therapy in HCC?

Considering the clinical and biological characteristics of HCC, patient enrichment therapeutic strategies or selection would be crucial to show a benefit in overall survival (OS) (Llovet and Hernandez-Gea, 2014).

The clinical outcomes of therapy in HCC could be improved by combining antiangiogenic agents and antiblastic chemotherapy (AC) and or other targeted molecules.

Combined treatments with bevacizumab plus CAPOX (capecitabine and oxaliplatin) or with bevacizumab plus GEMOX (gemcitabine and oxaliplatin) in HCC patients accounted in median survivals of less than 10 months (Zhu et al., 2006; Sun et al., 2011). On the basis of hopeful results from plus doxorubicin sorafenib in HCC, a phase III randomized trial (CALGB80802) comparing sorafenib alone vs. sorafenib plus doxorubicin is ongoing in patients with advanced HCC (Berretta et al., 2013). Other study combining sorafenib with GEMOX, modified FOLFOX (Folate, 5-FU and oxaliplatin), or CAPOX are ongoing.

Another approach has evaluated the join of antiangiogenic therapy with tyrosin Kinase inhibitors (TKI) of other targeted drugs. In a randomized phase II trial in advanced HCC, Tivantinib, a c-MET inhibitor, in comparison with placebo, demonstrated an augmented time to progression (TTP), particularly in tissue of patients bearing high MET expression (Santoro et al., 2013). Another example is Cabozantinib, a TKI of c-MET/VEGF receptor 2 (VEGFR2), is undergoing a phase III valuation in HCC patients who not tolerate or did failed treatment with sorafenib (Verslype et al., 2012). Everolimus, a m-TOR inhibitor, in a randomized phase III trial (EVOLVE-1) has been compared with best supportive care alone in the second-line treatment of HCC patients relapsed from sorefinib. Evenmore, no significant survival advantage has been demonstrated (Zhu et al., 2014a). Everolimus has been also used together sorafenib in a phase I trial; preliminary results shown a development of grade 3/4 thrombocytopenia in 43% of the HCC patients (Finn et al., 2013).

## Materials and methods

With the aim to evaluate the actual sceneries of angiogenesis inhibitors for the treatment of HCC, a search on the Cochrane Library and PubMed has been performed matching the key words “angiogenesis inhibitors treatment,” “target therapy,” and “HCC,” limited to the English written literature but with no restriction of time. Two authors (MB and RD) have examined the titles of 1328 papers retrieved.

Papers that did not include HCC series and/or case reports have been excluded, while ambiguous titles have been primarily included, leaving 240 articles. A search on abstracts or full text had led to the exclusion of other not pertinent papers. For trials performed by the same research institute at different times, the most recent and complete one has been included, unless different methods or endpoints or specific issues had been addressed. We also excluded the papers whose full text or at least abstract were not available. The references of important and pertinent papers have been searched for other relevant articles. At last 30 clinical trials have been retrieved fulfilling the requisite for analysis.

## Angiogenesis inhibitor drugs

In recent times, the exact identification of cellular signaling pathways playing a key role in the angiogenesis of HCC (Figure 1), has lead to the development of new molecules for targeted therapies (Table 1), (D'Amico et al., 2015a).

**Table 1:** **Main characteristics of angiogenesis inhibitor drugs**.

**Drugs**	**Typology**	**Targets**	**HCC therapeutic indication and ongoing study**
Bevacizumab	Monoclonal antibody	VEGF	Phase II study in combination with Erlotinib (NCT00881751)
Brivanib	Small molecule multikinase inhibitor	VEGFR, FGF	Advanced stage, Phase III study in patients who failed Sorafenib (NCT00825955), and first line (NCT00858871)
Cabozatinib	Small molecule multikinase inhibitor	c-MET, RET, VEGFR1-3, c-KIT,	Preclinical study, yet
Cediranib	Small molecule multikinase inhibitor	VEGFR 2, PDGFR, c-Kit	Advanced stage, phase II study
Everolimus	Small molecule	m-TOR	Not registered; phase III study did not show significant efficacy
Lenvatinib	Small molecule multikinase inhibitor	VEGFRs 1, 2 and 3, FGFRs 1 PDGFRα, RET, KIT	Not registered; phase III trial underway (NCT01761266)
Linifanib	Small molecule multikinase inhibitor	VEGFR 2, PDGFR families	Advanced stage, phase III study first-line vs. sorafenib (NCT01009593)
Nintedanib	Small molecule multikinase inhibitor	VEGFRs 1, 2 and 3, FGFRs 1 PDGFR	phase design study vs. sorafenib (NCT00987935 and NCT01004003)
Ramucirumab	Monoclonal antibody	Selective VEGFR 2	Not registered; phase III study with conflicting results
Refametinib	Small molecule mitogen-activated protein kinase inhibitor	MEK 1-2	Advanced stage, phase II study in patients who failed sorafenib (NCT01915589) and in combination with sorafenib (NCT01915602)
Regorafenib	Small molecule multikinase inhibitor	VEGFR1-3, c-KIT, TKI-like, EGF-like 2, PDGF 2, FGF 1, RET, RAF-1, BRAF, MAPK	Not registered; phase III study underway recruiting patients who progressed under sorafenib (NCT01774344)
Sorafenib	Small molecule multikinase inhibitor	VEGFR 2, PDGFR, c-Kit, BRAF	Advanced stage; not recommended for use in adjuvant treatment
Trebananib	Small molecule angiogenesis inhibitor	Tie-2 (Angiopoietin)	Not registered; phase II study no showed improvement of OS
Vatalanib	Small molecule angiogenesis inhibitor	VEGFRs 1, 2 and 3, PDGFR c-FMS	Advanced stage, phase II in combination with Doxorubicin

### Bevacizumab

The prototypical angiogenesis inhibitor, bevacizumab, an anti-VEGF monoclonal antibody directed against VEGF, is the first anti-angiogenesis agent to be approved as an antineoplastic drug. By neutralizing this ligand, bevacizumab prevents stimulation of VEGFR and reduces the angiogenesis. As described above, Bevacizumab given positive results both as a mono agent and in combination either with several antiblastics (Gemox, Folfox etc) or erlotinib in many phase II trials in advanced HCC patients. (Zhu, 2006; Thomas et al., 2009; Hsu et al., 2010; Sun et al., 2011). It should be noted, however, that bleeding is an uncommon but well-documented side effect of bevacizumab (Boige et al., 2012). In patients with HCC, such bleeding is particularly dangerous as they often have underlying coagulopathy secondary to liver dysfunction. A potential niche for bevacizumab may be as an adjunct to the Transarterial chemoembolization (TACE) procedure. In one clinical trial, this combination has shown to produce an impressive survival benefit without adding substantial toxicity (Buijs et al., 2013). A study of bevacizumab combined with erlotinib has reported 9.0 and 15.6 months in PFS and OS respectively, resulting in, an important improvement of 4 months PFS (Thomas et al., 2009). In addition, current phase II trial, also combining sorafenib vs. bevacizumab and erlotinib (NCT00881751) is ongoing.

### Brivanib

Brivanib is a TKI, with high specificity for both VEGFR and FGF receptor (FGFR) signaling pathways (Cai et al., 2008). It has shown hopeful anti-tumor activity in five of six patients resistant to sorafenib (Huynh et al., 2008; Park et al., 2011; Finn et al., 2012). Furthermore, in phase II trials for advanced HCC patients, Brivanib as first-line agent has not achieved the premeditated primary endpoint showing a 6 months PFS rate but established an OS of 10 months. Particularly, the 10 months OS has been higher than the 6.5 months OS in the Asia sorafenib trial (Park et al., 2011), but TTP resulting simlar (2.8 months). On the other hand, the numerous randomized phase III brivanib trials in HCC patients at risk (BRISK) performed to estimate the role of brivanib have also been unsatisfactory. In addition, in patients who progressed on/after or were intolerant to sorafenib the brivanib-post sorafenib (BRISK-PS) trial (NCT00825955) evaluating brivanib vs. placebo, had not achieved the primary endpoint of statistically improving OS (9.4 vs. 8.2 months, *P* = 0.3307) (Llovet et al., 2013). The BRISK-FL trial (NCT00858871) has compared the safety and efficacy of brivanib and sorafenib in patients with advanced/metastatic HCC who were naïve for other therapy. This investigation study has been also unsatisfactory but has showed non-inferiority for brivanib vs. sorafenib. In fact, in this study the primary endpoint in improving OS has failed (9.5 months brivanib vs. 9.9 months sorafenib), and secondary endpoints were alike in both study arms (Johnson et al., 2013).

### Cabozantinib

The cabozantinib s-malate (salt form), is a small molecule receptor TKI, bioavailable orally. This TKI drug binds to several TK receptors, which are often over expressed in a lot of cancer cell types, including hepatocyte growth factor receptor (MET), RET (rearranged during transfection), and simultaneously inhibits angiogenic factor receptor as VEGFR-1, VEGFR-2, and VEGFR-3. In addition an inhibition of mast/stem cell growth factor (KIT), FMS-like tyrosine kinase 3 (FLT-3), TIE-2 (TEK tyrosine kinase, endothelial), tropomyosin-related kinase B (TRKB), and AXL lead to tumor regression (Roy et al., 2015).

Antitumor effects of cabozantinib, a dual inhibitor of MET and VEGFR2, have been examined in cultured HCC cells as well as *in vivo* models. In the study of Xiang et al. (2014) phosphorylated MET (p-MET) has been measured in 29 resected HCC specimens, and have been correlated with response to sorafenib as post-surgery adjuvant therapy. The results have shown that high level of p-MET in resected HCC specimens have been associated with resistance to adjuvant sorafenib therapy. Cabozantinib has inhibited tumor growth in p-MET-positive and p-MET-negative HCC by decreasing angiogenesis, inhibiting proliferation, and promoting apoptosis. Notably, in the experimental metastatic mouse model, cabozantinib has reduced the number of metastasis in the lung and liver. They have concluded that patients with HCC carrying high level of p-MET are unresponsive to adjuvant treatment with sorafenib. The double blockade of VEGFR2 and MET by cabozantinib has significant antitumor activities in HCC, and the activation of MET in HCC may be a promising efficacy-predicting biomarker (Johnson et al., 2013).

### Cediranib

Cediranib (AZD2171) is an additional multitargeted inhibitor of VEGFR, PDGFRβ, FLT3 and c-KIT. In advanced HCC a phase II clinical trial, recorded a 5.8 months OS and 2.8 months TTP, and authors observed high occurrence of adverse events concluding that Cediranib was not successful at schedule and dosage of 45 mg/day (Alberts et al., 2012). A consequent phase II study with cediranib at a reduced dose (30 mg/day) demonstrated inefficient antineoplastic effectiveness in advanced HCC with a different acceptability profile. Results of the 5.3 months PFS and 11.7 months OS in this set were compared positively to data reported with 45 mg/day dosing of cediranib in advanced HCC (2.8 months TTP and 5.8 months OS). Prolongation of cure at a dose of 30 mg/day and patient set bias might have contributed to dissimilar outcome (Zhu et al., 2013a).

### Everolimus

Everolimus, a rapamycin analog, inhibits the mTOR pathway that is a key regulator of growth, angiogenesis and tumor cell survival. Since it is involved in hepatocarcinogenesis, m-TOR inhibition with everolimus has been investigated in a randomized, placebo-controlled Phase III trial (EVOLVE) in patients with Barcelona clinic liver cancer B/C HCC after failure of sorafenib treatment (Zhu et al., 2014b). Patients have been randomly assigned (2:1) to treatment with everolimus (*n* = 362, 7.5 mg QD) or placebo (*n* = 184). The primary end point OS (7.6 vs. 7.3 months; HR 1.05, 95% CI 0.86–1.27; *p* = 0.675) as well as the TTP (3.0 vs. 2.6 months; HR 0.93, 95% CI 0.75–1.15) have been similar in both arms. Thus, everolimus is considered to be inactive in patients with HCC (Llovet, 2014).

Another study focused on the PI3K/Akt/mTOR signaling cascade, as the activities of mTOR inhibitors and sorafenib, arise at separate stages along two pathways, their combination could be complementary and provide further successful suppression of HCC. Although the combination of sorafenib and everolimus have shown synergic inhibition of HCC. Further doubts relate to the most effective means of administering the drug combination and whether patients who have been unresponsive or intolerant to sorafenib could subsequently benefit from an mTOR inhibitor (Piguet et al., 2011).

### Lenvatinib

Lenvatinib an oral multi-TKI of VEGFRs 1–3, FGFRs 1–4, platelet-derived growth factor (PDGF) receptor alpha, and KIT and RET and KIT proto-oncogenes (Schlumberger et al., 2015). It was approved for radioiodine-refractory differentiated thyroid cancer, showing an impressive improvement in PFS (Schlumberger et al., 2015). The hazard ratio (HR) for progression or death was 0.21 with a 99% CI interval of 0.14 to 0.31 (*p* < 0.001) (Schlumberger et al., 2015). Additionally, nearly 2/3 of the patients (64.8%) showed an objective response (OR) to the lenvatinib treatment according to RECIST 1.1 criteria, including 4 complete responses. The most common treatment-related adverse events were similar to other TKIs (hypertension, diarrhea and fatigue). A total of 14% of the patients were at need to stop treatment with lenvatinib due to toxicity (Schlumberger et al., 2015). A further relevant safety issue with should be kept under surveillance in other trials investigating lenvatinib was the observation that 6 of 20 deaths that occurred during the treatment period were considered to be drug-related (Schlumberger et al., 2015). Currently, a Phase III trial is investigating the safety and efficacy of lenvatinib vs. sorafenib in the first-line setting (NCT01761266), for patients with advanced HCC.

### Linifanib

Linifanib (ABT-869), is a novel inhibitor of all VEGFR and PDGFR tyrosine kinases (Albert et al., 2006). In an open label, phase II trial, Linifanib as single agent demonstrated significant clinical activity (OS 9.7 months and TTP 5.4 months) in patients with advanced HCC (Toh et al., 2013). ABT-869 recorded a high acceptable safety profile in HCC patients. Current randomized phase III trial is ongoing in 1035 patients with advanced HCC who had no previous pharmacotherapy; it evaluates the effectiveness of linifanib as first-line therapy vs. sorafenib (NCT01009593). This trial has failed to account its primary end point, showing a similar OS between linifanib and sorafenib (9.1 months vs. 9.8 months respectively). TTP 5.4 months privileged linifanib vs. 4.0 months of sorafenib (Cainap et al., 2015).

### Nintedanib

Recently, an orally available TKI of VEGFR 1–3, PDGFR and FGFR Nintedanib (BIBF 1120) has been developed. BIBF 1120 evidently inhibits tumor development and angiogenesis in an *ex-vivo* model and exhibits moderately effects on HCC cell lines (Hilberg et al., 2008; Kudo et al., 2011; Tai et al., 2014). In patients with metastatic relapsed non-small cell lung cancer (NSCLC) and poor prognosis, who had failed first-line chemotherapy, eligible for phase II study (NCT00805194) shown remarkably benefit in median PFS and OS, by nintedanib-based therapy (Tai et al., 2014). A regimen for treatment of advanced ovarian cancer combining nintedanib with carboplatin and paclitaxel is ongoing (NCT01015118). Also in HCC patients, nintedanib is waiting to being compared in terms of the efficacy/safety with sorafenib (NCT01004003 and NCT00987935).

### Ramucirumab

Ramucirumab is a monoclonal antibody wholly humanized, designed to bind selectively to the extracellular domain VEGFR 2. This is a new therapeutic option as monotherapy or in combination with paclitaxel that has shown to improve OS in patients receiving second-line treatment for metastatic gastric cancer (Fuchs et al., 2014; Wilke et al., 2014). In addition, a phase II clinical trial in 42 patients with metastatic HCC has demonstrated encouraging anticancer effect of ramucirumab, showing 4 months median PFS and 12 months OS. The most of patients evaluated in this trial had a well conserved liver functions. An appealing result in this trial is the obtained OS stratified by liver function difference. In fact the authors reported a longer OS favoring ramucirumab in Child-Pugh B group than Child-Pugh A group (18.0 months vs. 4.4 months). Both groups are Barcelona clinic liver cancer-C (Zhu et al., 2013b). The phase III REACH study, comparing ramucirumab vs. placebo in patients with advanced HCC, after failure to sorafenib, missed its primary end point. In fact the OS in the intention-to-treat (ITT) patients (*n* = 565) has not been significantly diverse between the ramucirumab and the placebo group (HR 0.866, 95% confidence interval (CI) 0.717–1.046; *p* = 0.1391; median OS 9.2 months for ramucirumab vs. 7.6 months for placebo). Nevertheless, ramucirumab has resulted in a benefited PFS compared to placebo (HR 0.625, 95% CI 0.522–0.750; *p* < 0.001; median PFS 2.8 months for ramucirumab vs. 2.1 months for placebo) in the ITT population, without any safety concerns. However, in the subgroup of patients with baseline α-fetoprotein (AFP) 400 ng/ml (*n* = 250) the OS has been significantly longer for the patients treated with ramucirumab (HR 0.67, 95% CI 0.51–0.90; *p* = 0.0059) with a median OS of 7.8 and 4.2 months for ramucirumab and placebo respectively (Zhu et al., 2015). This data confirms that elevated value of AFP is a poor prognostic factor. The reason why ramucirumab is more efficacy in this setting is not clear, yet. Thus, ramucirumab will be developed further in the similar population with elevated AFP after sorafenib treatment progression or intolerance.

### Refametinib

The MAPK/ERK kinases (MEK) 1 and 2 are downstream components of the RAS signal transduction pathway, which is constitutionally active in the case of a Ras mutation. Pre-clinical studies indicate that activation of this pathway results in increased tumor growth and apoptotic resistance (Roberts and Der, 2007). Although mutations in the small GTPases of the Ras family are much less frequent in HCC in comparison to colorectal cancer (CRC) or pancreatic cancer, MEK inhibition seems to be an effective approach in preclinical HCC models (Schmieder et al., 2013). Refametinib (BAY 869766) is an allosteric inhibitor of MEK l/2 (Iverson et al., 2009). In a Phase II trial, 70 Asian HCC patients have been treated with a combination of refametinib and sorafenib (Lim et al., 2014). Four patients have shown an objective response (OR). Duration of response in these four patients has been 85, 128, 335, and 382 days, respectively. In three of these patients responding to refametinib and sorafenib, RAS mutations have been identified in plasma samples using the BEAMing technology. BEAMing technology compromises techniques such as PCR, hybridization and flow cytometry to amplify circulating tumor-derived DNA, and has the capacity to detect mutant DNA from or its fragments when present at ratios>1:10000 (0.01%) (Lim et al., 2014). The observation that patients with Ras mutations responded to the combination of refametinib and sorafenib have led to the rationale to further explore refametinib in combination with sorafenib in patients with HCC harboring a Ras mutation. Refametinib is currently investigated in two Phase II trials either as monotherapy in patients with advanced HCC who failed sorafenib treatment as well as in combination with sorafenib in the first-line treatment of advanced HCC (NCT01915589, NCT01915602).

### Regorafenib

Regorafenib is an oral novel diphenylurea multikinase inhibitor of VEGFR1–3, c-KIT, tyrosine kinase with immunoglobulin-like and EGF-like domains-2, PDGFR-2, Fibroblast growth factor receptor-1, RET, RAF-1, BRAF, and p38 MAP kinase (Strumberg and Schultheis, 2012). Although it is structurally similar to sorafenib, the addition of a fluorine atom in the central phenyl ring might result in a higher efficacy. It has been approved for the treatment of metastatic Colorectal cancer after failure to oxaliplatin and irinorecan-based systemic chemotherapy showing a significant improve of OS compared to placebo arm (Strumberg and Schultheis, 2012). Moreover, it has also been shown to be effective for the treatment of metastatic gastrointestinal stroma tumors (GIST) after failure of imatinib and sunitinib (Demetri et al., 2013). A multicenter, open label phase II study in intermediate or advanced HCC patients who failed to sorafenib has been reported showing a signal for activity (Bruix et al., 2013). The side effect profile of regorafenib seems acceptable and similar to sorafenib. Currently, a Phase III trial (RESORCE) of regorafenib in patients who progressed under sorafenib treatment is ongoing (NCT01774344).

### Sorafenib

Sorafenib is an oral multi-kinase inhibitor that suppresses tumor neo-angiogenesis and proliferation inhibiting the TK activities of VEGFR 1–3 and of PDGFR-II. Moreover, it also inhibits the serine-threonine kinase RAF-1 and B-Raf. The efficacy of sorafenib has been demonstrated in two randomized controlled trials. For convenience we report the data on SHARP trial, where sorafenib has shown an improvement in OS (10.7 and. 7.9 months in the treatment and placebo arm respectively) and good safety in patients with HCC, making sorafenib the standard of care for the treatment of patients with advanced HCC (Llovet et al., 2008). The high relapse rate of HCC after surgical resection and/or local ablation has been the rational to start the STORM trial, which tested adjuvant treatment with sorafenib vs. placebo after R0 resection or complete ablation of HCC. Unfortunately, sorafenib didn't' improve in PFS in this Phase III trial and at some time has been associated with substantial side effects comparable to adverse events reported in advanced HCC patients (Bruix et al., 2014b). Therefore, adjuvant treatment with sorafenib should not be suggested in patients with HCC after surgical resection and/or local ablation.

#### Sorafenib combined with transarterial chemoembolization (TACE)

Even though initial responses to sorafenib, the situation of advanced HCC treatment is still low and the majority of HCC patients account loss of efficacy, with poor prognosis and less than 1 year of survival (Berretta et al., 2015b). Conventional TACE (cTACE) is a treatment able to improve (Lo et al., 2002; Llovet et al., 2003) survival, with rates of 75%, 47% and 26% at 1, 2 and 3 years respectively (Lo et al., 2002). Drug eluting bead (DEB)-TACE is an evolution of cTACE in drug delivery to raise loco-regional drug concentration (Hong et al., 2006; Varela et al., 2007). It appears to notably go beyond the antiblastic efficacy of TACE, with higher response rates ranging from 70 to 80%, while attenuation the adverse events (Reyes et al., 2009; Lammer et al., 2010). A high prevalence of relapse is a restriction of TACE, likely due to the up regulation of VEGF and PDGFR, which sequentially increases tumor angiogenesis; therefore, the combination of TACE with antiangiogenic drugs has stranded out as an enhancement, aiming to decrease post-TACE angiogenesis and the incidence of metastatic disease and, as much as possible, improving loco-regional treatment efficiency. A clinical trial with sorafenib joint with DEBTACE (phase II study) in patients affected by advanced HCC has shown a significant efficacy, 58% OR, about to 100% DCR, and tumor size reduced by 4% (from 6.0 to 5.8 cm; *P* = 0.05) after 1 combined therapy cycle (Pawlik et al., 2011). Quite a few clinical trials have also demonstrated promising results for combination targeted agents with TACE. One prospective non-randomized controlled trial comparing the efficacy of sorafenib in combination with TACE or TACE alone in unresectable or advanced HCC revealed that the coactions of sorafenib prolonged TTP (6.3 months vs. 4.3 months; *P* = 0.004) and the median OS (7.5 months vs. 5.1 months; *P* = 0.009) (Bai et al., 2013). Another retrospective multicenter study on 222 patients affected by advanced HCC showed antitumor efficacy, with a 12 months OS and 8.5 months TTP for the combination of sorafenib and TACE. Promising, sorafenib in combination with TACE seems to be a powerful treatment for patients affected by advanced HCC (Zhao et al., 2013).

#### Sorafenib combined with antiblastic chemotherapy (AC) or other targeted molecules

In several studies sorafenib decreased tumor size less clearly when compared with placebo. However, AC shrinks the proper dimensions of tumor, despite the lack of compelling proof in benefiting survival for advanced HCC patients. Consequently, several phase II/III clinical trials have been started worldwide to evaluate sorafenib plus combination to sorafenib as monotherapy (Sun et al., 2011). Sadly, the “sorafenib plus” combination didn't demonstrate advantage in clinical trials. The SEARCH trial, sorafenib with erlotinib combination, a phase III study (NCT00901901), showed no survival improve (OS 9.5 months vs. 8.5 months, *p* = 0.204), according to the study reported at the Congress in Vienna in 2012 by European Society for Medical Oncology (ESMO).

### Trebananib

Angiopoietins are vascular growth factors, binding to the Tie-2 receptor. Angiopoietin-1 mediates vessel maturation, adhesion, migration and survival, whereas angiopoietin-2 promotes cell death and disrupts vascularization. Trebananib (AMG386) is an antiangiogenic compound that sequesters both angiopoietin-1 and -2, thus preventing their interaction with the Tie-2 receptor. To investigate the safety and efficacy of trebananib plus sorafenib 60 patients affected by advanced HCC were treated in a two-cohort, uncontrolled Phase II trial (cohort 1: Trebananib IV at 10 mg/kg weekly; cohort 2: Trebananib IV at 15 mg/kg weekly) (Abou-Alfa et al., 2014). The primary end point was PFS at 4 months. Although, trebananib at both dose levels plus sorafenib was well tolerated, the study did not show an improvement in PFS at 4 months compared to a historical control. However, the median OS in the 10 mg/kg cohort was 17 months, and the PFS was 9 months. Currently, no further trial of trebananib in HCC is planned, since the ovarian Phase III trial (TRINOVA-1) did not show an improvement in OS (secondary end point) (Monk et al., 2014).

### Vatalanib

Vatalanib (PTK787), is a TKI that binds VEGFR by blocking ATP-binding sites; it also inhibits both FLT-1 and Flk-1/KDR and other class III TKr, such as PDGFR-II, FLT-4, c-kit, and c-fms (Wood et al., 2000). A phase I/II research of vatalanib combined with intravenous doxorubicin in advanced HCC was conducted, reporting a 5.4 months PFS and 7.3 months OS. This was the earliest trial combining TKI and doxorubicin that established strong efficacy in advanced HCC patients and provided the root for planning future trials combining anti-angiogenic agents and AC to improve vatalanib effectiveness (Yau et al., 2010). A preclinical study reported that the synergic activity of Vatalinib and interferon/Fluorouracil allowing noticeably monitoring of tumor development both in cell lines and a xenograft HCC model (Murakami et al., 2011).

## Conclusion

The angiogenesis is a dynamic essential process playing a critical role in tumor growth and metastatic diffusion. The development of new prognostic factors, tumor markers, imaging techniques, and therapeutic modalities is the consequence of the understanding the basic principles of the biology of angiogenesis. Prognostic angiogenesis biomarker (i.e., VEGF level) plays a key role in the circumstance of several cancer diseases.

Blocking angiogenesis represents an effective approach to HCC. Current clinical and preclinical trials propose the benefit of an arrangement of anti-angiogenic drugs with AC, radiotherapy or other targeted drugs in several malignancies. Nevertheless, in order to choose the optimal and most effective combination of drugs (AC and anti-angiogenic), it is indispensable to knowledge the mechanisms by which anti-angiogenesis effects could be obtained. Validated molecular biomarkers, tissue histotype, imaging and genetic could be integrated routinely into preclinical and clinical trials, in order to choose the best possible scheduling of these drugs, especially in the so called “frail patients” (Berretta et al., 2011; Nunnari et al., 2012).

Unfortunately insufficient data are reported, also in clinical trials, in so called “disadvantages” patients (HIV positive and elderly) who represent a growing setting in the HCC “scenario” (Nunnari et al., 2012; Di Benedetto et al., 2013a; Berretta et al., 2015a).

We know that also in these patients the standard approach used in the general population is safe and efficacy (Di Benedetto et al., 2008, 2011, 2012, 2013b; Ursino et al., 2012; D'Amico et al., 2015a,b; Berretta et al., 2015c).

Finally, we think that the recent progresses in genetics have provided good opportunities to identify prognostic factors and predictive markers of efficacy of antiangiogenic treatments. Moreover, genetic markers could be a tool to identify responsive patients to targeted therapies, and exclude patients at high risk to develop severe adverse events. (De Monaco et al., 2014).

In the future, we believe that the right features of these challenges are based on a multidisciplinary treatment approach, in order to rationalize the costs of these treatments due to aimed-interventions (Berretta et al., 2014).

## Author contributions

MB, LR, and RD wrote and organized the manuscript. FD, AL, VD, GF, and PD performed the data collection.

### Conflict of interest statement

The authors declare that the research was conducted in the absence of any commercial or financial relationships that could be construed as a potential conflict of interest.
